# Next-Generation Sequencing-based genomic profiling of brain metastases of primary ovarian cancer identifies high number of BRCA-mutations

**DOI:** 10.1007/s11060-017-2459-z

**Published:** 2017-05-11

**Authors:** S. Balendran, S. Liebmann-Reindl, A. S. Berghoff, T. Reischer, N. Popitsch, C. B. Geier, L. Kenner, P. Birner, B. Streubel, M. Preusser

**Affiliations:** 1 Department of Pathology, Medical University of Vienna, Waehringer Guertel 18-20, 1090 Vienna, Austria; 2 Core Facility Genomics, Medical University of Vienna, Vienna, Austria; 3 Department of Medicine I, Medical University of Vienna, Vienna, Austria; 4 Department of Obstetrics and Gynecology, Medical University of Vienna, Vienna, Austria; 5 Children’s Cancer Research Institute, St. Anna Kinderkrebsforschung, Vienna, Austria; 6 Immunology Outpatient Clinic, Schwarzspanierstraße 15/1/9, Vienna, Austria; 7 Comprehensive Cancer Center, Central Nervous System Unit (CCC-CNS), Medical University of Vienna, Vienna, Austria; 8 Neurology Clinic, Heidelberg University Medical Center and Neurooncology Program, National Center for Tumor Diseases Heidelberg, Heidelberg, Germany; 9 Department of Laboratory Animal Pathology, University of Veterinary Medicine Vienna, Vienna, Austria; 10 Ludwig Boltzmann Institute for Cancer Research LBI-CR, Vienna, Austria

**Keywords:** Brain metastases, Ovarian cancer, Next-Generation Sequencing, *BRCA1*/*2*, *TP53*, *ATM*

## Abstract

Ovarian cancer represents the most common gynaecological malignancy and has the highest mortality of all female reproductive cancers. It has a rare predilection to develop brain metastases (BM). In this study, we evaluated the mutational profile of ovarian cancer metastases through Next-Generation Sequencing (NGS) with the aim of identifying potential clinically actionable genetic alterations with options for small molecule targeted therapy. Library preparation was conducted using Illumina TruSight Rapid Capture Kit in combination with a cancer specific enrichment kit covering 94 genes. *BRCA*-mutations were confirmed by using TruSeq Custom Amplicon Low Input Kit in combination with a custom-designed *BRCA* gene panel. In our cohort all eight sequenced BM samples exhibited a multitude of variant alterations, each with unique molecular profiles. The 37 identified variants were distributed over 22 cancer-related genes (23.4%). The number of mutated genes per sample ranged from 3 to 7 with a median of 4.5. The most commonly altered genes were *BRCA1*/*2, TP53*, and *ATM*. In total, 7 out of 8 samples revealed either a *BRCA1* or a *BRCA2* pathogenic mutation. Furthermore, all eight BM samples showed mutations in at least one DNA repair gene. Our NGS study of BM of ovarian carcinoma revealed a significant number of *BRCA*-mutations beside *TP53, ATM* and *CHEK2* mutations. These findings strongly suggest the implication of BRCA and DNA repair malfunction in ovarian cancer metastasizing to the brain. Based on these findings, pharmacological PARP inhibition could be one potential targeted therapeutic for brain metastatic ovarian cancer patients.

**Electronic supplementary material:**

The online version of this article (doi:10.1007/s11060-017-2459-z) contains supplementary material, which is available to authorized users.

## Introduction

Ovarian cancer (OC) represents the most common gynaecological malignancy and has the highest mortality in the developed world [1]. The vast majority of OCs are of epithelial origin [2] and the most frequent subtype of epithelial OCs is the serous carcinoma [3]. More than 70% of OCs are diagnosed in advanced stages with detectable transperitoneal spread [4]. The 5-year survival rate is approximately 30% [5]. Despite progress in the diagnosis and treatment of these tumors, the rates of relapse and distant metastasis remain high [1]. Given the high recurrence rate and poor long-term survival of women with advanced stage disease, there is a strong need to document the unique metastatic patterns of epithelial OC by comparing the differences in genetic profiles between primary and metastatic tissue specimens [6].

Clinical observation and retrospective clinical studies suggest that serous OCs grow very efficiently within the peritoneal cavity, but rarely metastasize beyond it [7]. Accordingly, brain metastases (BM) from OC is a very rare phenomenon and a late-stage disease manifestation, usually occurring in the context of widely disseminated disease [1]. The incidence of BM from epithelial OC ranges from 0.29 to 5% [8, 9]. However, the incidence of BM seems to be increasing (11.6%) since the introduction of platinum-based chemotherapy [10] and the more frequent use of sensitive detection methods [11]. BM are associated with a large burden of neurological symptoms and a poor survival prognosis [12]. Treatments vary widely, including chemotherapy, steroids, whole brain radiation therapy, surgical resection, and stereotactic radiosurgery [13].

To date, most research efforts have focused on defining the molecular characteristics of primary OC [6, 14, 15]. Only very limited data are available on molecular aberrations in BM of OC [16–18]. In this study, we evaluated the mutational profile of OC metastases through Next-Generation Sequencing (NGS) with the aim of identifying potential clinically actionable genetic alterations with options for small molecule targeted therapy.

## Methods

### Patients

Only BM of ovarian carcinomas were included into this retrospective observational study. In total, ten ovarian cancer BM of the same number of patients were identified and subsequently investigated by NGS. All patients were female with a median age of 51 years (range 36–65 years) at diagnosis of ovarian cancer. The median interval between primary diagnosis of ovarian cancer and diagnosis of BM was 30.5 months (range 0–61 months). Nine of the ten patients were treated with a platinum-based chemotherapy before the diagnosis of BM. Supplementary Table S1 shows further patient´s baseline characteristics. For histological subtypes, nine were serous carcinoma and one small cell carcinoma of the ovary.

### Publicly available datasets

Genomic mutational profiles of ovarian serous cystadenocarcinoma were obtained from the cBioPortal for Cancer Genomics (http://www.cbioportal.org) [19, 20]. This TCGA provisional dataset is directly from the TCGA data center and consists of 316 sequenced tumors of clinically annotated stage-II–IV high-grade serous ovarian cancer. TCGA performed exome capture and massively parallel sequencing on the Illumina GAIIx platform (236 samples) or ABI SOLiD 3 platform (80 samples) [14].

### Illumina sequencing panel

#### Illumina TruSight Rapid Capture Kit

Genomic DNA was isolated from formalin fixed, paraffin embedded (FFPE) tissue sections using QIAamp DNA FFPE Tissue Kit (Qiagen) according to the user manual. Quantification was performed with Qubit^®^ dsDNA HS Assay Kit (Invitrogen) according to the instructions provided by the manufacturer. The quality of DNA samples was controlled prior to sample selection for NGS using a qPCR-based assay. Library preparation was conducted using TruSight Rapid Capture Kit in combination with a cancer-specific enrichment kit covering 94 genes provided by Illumina Inc. (Supplementary Table S2). Subsequently 150 bp paired-end sequencing was performed on a MiSeq System (Illumina, USA) with reagent kit v2. The read alignment and variant calling were performed with BaseSpace BWA Enrichment v1.0 App. The sequence is aligned with BWA Genome Alignment Software and variant calling is performed with GATK using the human reference sequence hg19/GRCh37. These variants were then annotated using the Illumina VariantStudio data analysis software.

A number of steps were used to filter nucleotide variants identified in the screening: First, intronic variants and synonymous SNVs were excluded. Subsequently, variants annotated in dbSNP without pathogenic relevance for the clinical phenotype were removed. The remaining variants were further filtered by excluding all variants that are not annotated as “pathogenic” or “likely pathogenic” by in silico prediction tools. This set was further filtered by excluding all variants showing a poor quality (read depth <30; alternative variant frequency <5). Manual and thorough observation of the variants was performed to exclude false variants. The resulting filtered sets of all eight samples were then combined to a total of 37 variants.

#### TruSeq Custom Amplicon Low Input Kit

Based on the presented results we aimed to focus on the analysis of both *BRCA* genes of interest and therefore used the TruSeq Custom Amplicon Low Input Kit (Illumina) in combination with a custom-designed *BRCA* gene panel. Selected FFPE samples were subjected to dual-pool amplicon-based library preparation. Subsequent sequencing of pooled libraries was performed on the MiniSeq sequencing platform using MiniSeq High Output Reagent Kit for 300-cycles.

Paired-end sequencing resulted in average 6115644.86 (6.1 Mio) paired-end passed filter reads per sample and mean amplicon coverage of 6774. Data analysis was conducted using on board Amplicon DS pipeline. Sequencing data was aligned to the reference genome UCSC hg19 using banded Smith–Waterman algorithm and variant calling was performed with Illumina Somatic variant caller. Filtering of all datasets was conducted manually according to predefined (custom) criteria.

### Statistical analysis

The statistical analysis was performed with SPSS (19.0). Mainly descriptive statistic was used. For association analyses with clinical data, Spearmen Correlation and Mann Whitney *U* test was applied. Due to the observational and hypothesis generating character of this study, we did not adjust for multiple testing [21]. The threshold for statistical significance was set at a p-value of less than 0.05.

## Results

### NGS mutational profile

#### Somatic mutations in OC BM

In two of the total ten cases, data quality was low, probably due to the fact that DNA was isolated from FFPE material and may have been degraded. These were excluded from further analysis. The remaining eight samples were successfully sequenced. The mean count of total aligned reads obtained per sample was 2.88 million (range 1.88–6.39 million) and the minimum sequencing depth was 543.5X.

Overall, 37 variants were detected with known or likely pathogenic effect. A total of 3231 variants were excluded because they were known polymorphisms, likely benign variants or did not pass the quality criteria specified in the Supplementary Methods section. The number of gene mutations per sample ranged from 3 to 7 with a median of 4.5. Three samples (37.5%) showed 3 mutations, one sample (12.5%) showed 4 mutations, two samples (25%) showed 5 mutations, and two samples (25%) showed 7 mutations (Table 1). The 37 variants consisted of 6 frameshift variants, 2 splice variants, another 3 were stop mutations, and the remaining 26 were missense variants (Table 1). In addition, base transitions (purine–purine and pyrimidine–pyrimidine) were considerably more frequent (61.29%) than transversions (purine–pyrimidine, vice versa) (Supplementary Figure S3). In our cohort all eight sequenced BM samples exhibited a multitude of variant alterations, each with unique molecular profiles. A few samples showed common mutations, however, each sample exhibited different overall molecular profiles. In summary, 20 of 37 variants are known COSMICs (catalogue of somatic mutations in cancer) and the remaining 17 were considered potentially biologically significant.

**Table 1:** Mutation profile of the eight successfully sequenced BM samples

Patient	Sample ID	Gene	Codon change	Variant frequency (%)	Amino acid change	COSMIC ID	Consequence	Confirmational BRCA-analysis
1	N32-08	BRCA1	c.5450C>A	6.6	p.Ser1817Ter		Stop gained	
		GPC3	c.359G>A	42.9	p.Arg120His		Missense variant	
		PRF1	c.272C>T	11.8	p.Ala91Val		Missense variant	
		SMAD4	c.906G>T	6.8	p.Trp302Cys	COSM1389045	Missense variant (splice region)	
		TP53	c.829T>G	62.9	p.Cys277Gly	COSM45074	Missense variant	
2	N93-95	BRCA1	c.3018_3021delTTCA	86.4	p.His1006GlnfsTer17		Frameshift variant (truncation)	
		CHEK2	c.1246A>G	13.1	p.Lys416Glu		Missense variant	
		NBN	c.1870C>T	8.4	p.Arg624Cys	COSM1102335	Missense variant	
		RB1	c.2104C>A	70.4	p.Gln702Lys	COSM76180	Missense variant (splice region)	
		TP53	c.376-2A>G	80.1	Splice mutation	COSM45672	Splice acceptor variant	
3	N423-99	CHEK2	c.1246A>G	12.6	p.Lys416Glu		Missense variant	
		FH	c.1384C>G	9.7	p.His462Asp		Missense variant	
		KIT	c.2540C>T	6.4	p.Thr847Met	COSM1212555	Missense variant	
4	N459-08	BRCA1	c.672_4096del^a^	99.9	Deletion		Gross deletion	√
		NF1	c.1154G>A	9.8	p.Arg385His	COSM133082	Missense variant	
		TP53	c.797G>T	91.8	p.Gly266Val	COSM10958	Missense variant	
5	N714-02	ALK	c.2763C>G	20	p.Phe921Leu		Missense variant	
		BRCA1	c.2679_2682delGAAA	66	p.Lys893AsnfsTer106		Frameshift variant (truncation)	√
		HNF1A	c.79A>C	50	p.Ile27Leu	COSM430522	Missense variant	
6	N926-06	ATM	c.5557G>A	48.3	p.Asp1853Asn	COSM41596	Missense variant	
		BRCA1	c.5329dupC	67.6	p.Gln1777ProfsTer74		Frameshift variant (elongation)	√
		MET	c.3029C>T	37.9	p.Thr1010Ile	COSM707	Missense variant	
		NBN	c.633T>A	65	p.Asp211Glu		Missense variant	
		RUNX1	c.508 + 1G>A	28.6	Splice mutation	COSM24722	Splice donor variant	
		SUFU	c.1128G>C	27.8	p.Glu376Asp		Missense variant	
		TP53	c.743G>A	23.8	p.Arg248Gln	COSM10662	Missense variant	
7	N1210-00	ATM	c.5188C>T	8.3	p.Arg1730Ter	COSM172204	Stop gained	
		BRCA2	c.3846_3847delTG	76	p.Val1283LysfsTer2		Frameshift variant (truncation)	√
		EPCAM	c.421A>G	41	p.Thr141Ala		Missense variant	
		FANCG	c.890C>T	93.9	p.Thr297Ile	COSM150601	Missense variant	
		PTCH1	c.1322G>T	7.1	p.Arg441Leu	COSM95099	Missense variant	
		RB1	c.1666C>T	10.6	p.Arg556Ter	COSM888	Stop gained	
		TP53	c.797G>A	79	p.Gly266Glu	COSM10867	Missense variant	
8	N1250-00	ATM	c.3161C>G	55	p.Pro1054Arg	COSM21827	Missense variant	
		BRCA2	c.5946delT	68.2	p.Ser1982ArgfsTer22	COSM26515	Frameshift variant (truncation)	
		CDKN2A	c.442G>A	48.2	p.Ala148Thr		Missense variant	
		TP53	c.428_429dupTG	71	p.Gln144CysfsTer27		Frameshift variant (elongation)	

^a^100% loss of heterozygosity of c.4039A>G (Exon 11b) was found in BM compared to heterozygous normal tissue

#### Mutated genes in OC BM

In a next step we searched for genes that were recurrently mutated in our cases. We tested 94 genes, and 72 genes displayed no alterations (Supplementary Table S2). The 37 identified variants were distributed over 22 genes (23.4%). The number of mutated genes per sample ranged from 3 to 7 with a median of 4.5, identical with the number of mutations. Just two of the 37 revealed mutations were known cancer hotspots: TP53 p.G266V and TP53 p.R248Q, the latter actually in OC [22], and TP53 p.R248Q mutation has been reported to aggregate and to be associated with metastasis [23]. The most commonly altered genes were *TP53* and *BRCA1* with 6/8 and 5/8 events, respectively. Aberrations in *ATM* were found in 3/8 samples. *BRCA2, CHEK2, NBN* and *RB1* showed aberrations in 2/8 cases, each. Furthermore, 15 other genes showed aberrations, however less frequently (n = 1) (Table 1).

The most common combination was *TP53* and *BRCA1* mutations (n = 4), followed by *TP53* and *ATM* (n = 3), and *TP53* and *BRCA2* (n = 2). The number of mutations was not associated with age (*p* > 0.05, Spearmen Correlation). There was a weak association between the tumor stage and the number of genomic abnormalities in BM, which did not reach statistical significance (*p* > 0.05, Mann Whitney *U* test). A trend in longer overall survival was seen in *BRCA1, BRCA2* and *TP53* mutation carriers compared with non-carriers, whereas just 1/8 patients revealed no mutations in these 3 genes and in addition showed a short overall survival. In summary, 7 out of 8 BM samples revealed either a *BRCA1* or a *BRCA2* pathogenic mutation. Furthermore, all eight BM samples showed mutations in at least one DNA repair gene (*BRCA1*/*2, ATM, CHEK2*) (Table 1).

### Genomic profile of ovarian serous cystadenocarcinoma (TCGA dataset)

Due to the lack of paired samples, i.e. corresponding primary tumor biopsies (PT) to each of our BM samples, we analyzed a publicly available dataset encompassing 316 ovarian serous cystadenocarcinoma (TCGA, Provisional) to be able to obtain at least a comparison between PT and BM. We focused mainly on the 94 genes in this TCGA dataset, which we analyzed in our BM samples (Supplementary Table S2). Overall, 7/22 genes, which were mutated in the BM samples, showed no alterations in the PM, whereas the remaining 15 genes were mutated as well. Notably, in agreement with the BM samples, *TP53* (86.71%), *BRCA1* (3.48%), and *BRCA2* (3.48%) were the most frequently altered genes. Additionally, *NF1* (4.43%), *RB1* (2.85%), and *KIT* (2.22%) were frequently mutated in the PT in contrast to the BM samples. Furthermore, in the PT samples *BRCA1* and *BRCA2* mutations were mutually exclusive. This is in agreement with our BM samples and with the findings of Mafficini et al. [24]. In addition, the ratio of *BRCA1* (11/316) to *BRCA2* (11/316) was balanced in the PT samples, whereas *BRCA2* (2/8) was less commonly mutated in the BM samples than *BRCA1* (5/8). The most common combination of gene alterations in the PT samples was *TP53* and *NF1* (n = 11), followed by *TP53* and *BRCA2* (n = 9), and *TP53* and *BRCA1* (n = 8). Analysis of the remaining 72/94 genes, which were not mutated in the BM samples, revealed that 43/72 were altered in the PT dataset, amongst which *EGFR* (2.22%), *APC* (2.2%), and *SMARCB1* (1.58%) have to be highlighted. When focusing on the 94 genes analyzed in the BM samples, we found the median number of gene mutations per PT sample and median number of mutated genes to be 1.0 (range 0–5).

### Confirmation of *BRCA-*mutations in four patients by using the TruSeq Custom Amplicon Low Input Panel

For conformational analysis in addition to BM samples, primary tumor and/or normal FFPE tissue samples were fortunately available in some cases. We were able to confirm *BRCA*-mutations in four patients using the TruSeq Custom Amplicon Low Input Kit. We validated the *BRCA*-mutations in patients #5 and #7, but no additional patient-matched tissues were available for testing of the mutational origin. We tested available patient-matched normal tissue additionally to BM in patient #6. In this case, the *BRCA1* mutation was found only in the tumor, thus confirming that this mutation was of somatic origin. Furthermore, the BRCA1 mutation p.Gln1777ProfsTer74 was already described in the context of OC by Mafficini and colleagues [24]. We tested PT and normal tissue additionally to BM in patient #4. We detected c.4039A>G (p.Arg1347Gly) in all tissues including normal tissue and concluded that this missense mutation is a germline mutation. The c.4039A>G is a variant of uncertain significance according to ACMG guidelines (RCV000034747.1). Interestingly the c.4039A>G was detected in all tissues at different frequencies: normal tissue showed 50%, PT revealed 75% and BM showed 100%. Thus, we conclude that the loss of heterozygosity/*BRCA1*-deletion is a somatic mutation and occurred in part of the PT sample and in the entire BM sample. In summary, all BRCA-mutations were confirmed by an independent analysis, and in two cases we could furthermore prove-due to additional normal tissue-that they were of somatic origin.

## Discussion

To the best of our knowledge, we present here for the first time results of NGS in BM of OC. Too little is currently known about the genomic makeup of OC BM to offer a potential pathway for targeted therapeutics in this disease. Because of the rarity of BM of OC, the number of studied patients in general as well as in our cohort has been small. With this NGS-based study, we aimed to get some insight for a better understanding of this rare phenomenon. We successfully sequenced eight BM samples of primary OC and detected 37 variants in total, distributed over 22 cancer-related genes (23.4%). Mutations per analyzed BM sample ranged from 3 to 7 with a median of 4.5. The most commonly altered genes were *BRCA1*/*2, TP53, ATM*, and among others, *CHEK2*. Consistently, *TP53, BRCA1*, and *BRCA2* were the most frequently altered genes in the TCGA ovarian serous cystadenocarcinoma data. Moreover, in line with other studies on the pattern of somatic mutations in human cancer genomes [15, 25], we observed a higher rate of transitions than transversions. Even if metastatic tumor spread is a very complex process consisting of many different events, the mutational spectrum of our BM specimens was surprisingly simple, which is consistent with the findings of Beltrame et al., who reported that the genomic architecture of relapsed disease was less heterogenous than that of the primary disease [26]. Nevertheless, no two BM shared an identical genetic profile, which is similar to published data about metastatic breast cancer [27, 28].

One of our major findings was the unexpectedly high number of *BRCA1*/*2* mutations in BM of OC (7 out of 8). The National Genome Atlas study identified somatic *BRCA1* and *BRCA2* mutations as a significant feature of high grade serous OC [14], but the characterized mutational frequencies were much lower [2, 20]. The *BRCA1* gene is involved in DNA repair, cell-cycle checkpoint control, chromatin remodeling, transcriptional regulation and mitogenesis, while the *BRCA2* has an important role in homologous recombination [29]. In our BM samples, 2/8 revealed a *BRCA2* mutation. Koul and colleagues hypothesized that *BRCA1* might have a possible role in ovarian tumors metastasizing to the brain [30], which is mostly in line with our mutational results. In our cohort, 5/8 patients showed a *BRCA1* mutation and 2/8 a *BRCA2* mutation. Patient #4 was an interesting case, because we had PT and normal tissue available in addition to BM. A *BRCA1*-deletion occurred in part of the PT sample and in the entire BM sample, suggesting that there was a positive selection of *BRCA1* mutation in BM compared to PT. In conclusion, mutated *BRCA1* and *BRCA2* seem to be important in the development of BM.

It was not surprising that *TP53* and *ATM* were mutated in our BM samples of primary OC. Previous studies have highlighted that *TP53*, that encodes the tumor suppressor protein p53, is the most frequently altered gene in serous OC [2, 17]. In addition, the majority of our genetic alterations identified in *TP53* were predicted to be deleterious, and 2 of the BM mutations were known hotspot mutations (p.G266V and p.R248Q) (http://cancerhotspots.org/). Based on these findings one can hypothesize that *TP53* plays a key role in BM specimens of primary OC. In our samples mutations in *TP53* and *ATM* (n = 3), and *BRCA1*/*2* and *ATM* (n = 3) are common combinations. ATM is a major regulator of DNA damage detection and repair [31]. In response to DNA damage, ATM controls the initial phosphorylation of a wide variety of downstream proteins such as TP53 and BRCA1 [32]. Moreover, ATM mutations can lead to deficiencies in DNA repair, which in return may give rise to cancer [33]. Therefore, it can be hypothesized that *ATM* does not promote OC metastasizing to the brain isolatedly but through the DNA damage-recognition and repair pathway together with *TP53* and *BRCA1*.

The mutation frequencies for *NF1, RB1*, and *KIT* in our BM samples were not in the range described for ovarian cancer specimens, they were less frequent. *EGFR* (2.22%), *APC* (2.2%), and *SMARCB1* (1.58%) have to be stressed here as well, because they were mutated in the TCGA dataset, but not in our BM samples. Beltrame and colleagues reported that somatic mutations showed a low rate of concordance between primary and recurrent disease in stage III–IV epithelial ovarian cancer, which may explain both these phenomena, or may be due to an underrepresentation of the mutational burden in the BM samples because of the small sample size [26].

In this study, we focused on identifying potentially actionable somatic mutations in metastatic OC. In total, 7/8 BM samples analyzed showed *BRCA1*/*2* mutations and, moreover, all 8 samples revealed mutations in at least one DNA repair gene (*BRCA1*/*2, ATM, CHEK2*). It is known that cells which are BRCA deficient and then undergo Poly (adenosine diphosphate-ribose) polymerase (PARP) inhibition (PARPi), suffer cell death [34]. Accordingly, Mateo and colleagues concluded that, if a cell was lacking homologous repair capacity because of *BRCA1, BRCA2* or *ATM* dysfunction or loss, then PARPi would lead to synthetic lethality due to cell cycle arrest and subsequent apoptosis [35]. Additionally, McCabe et al. showed via in vitro studies that, among others, ATM and CHEK2 abnormalities resulted in sensitivity to PARPi, suggesting that PARPi would be beneficial for a variety of genes involved in the DNA damage response [36]. The PARPi Olaparib has shown significant clinical activity in BRCA-mutated platinum-sensitive recurrent serous OC and has been approved for clinical use [37–40]. Based on these facts and on our findings, pharmacological PARPi could be one potential targeted therapeutic for brain metastatic OC patients, especially because PARPi Olaparib has shown evidence of crossing the blood–brain barrier [41]. Multiple PARP inhibitors are already at different stages of clinical development for the management of OC [42, 43].

Metastatic OC remains largely incurable, and thus a larger population based study and molecular genetic analyses of OCs metastatic to the brain are needed for a better understanding of the role of key genes like *BRCA1*/*2, TP53, ATM*, and *CHEK2* in this rare phenomenon. Because of the rarity of OC BM our sample size is relatively small. Therefore, the acquisition of sufficiently large cohorts consisting of matched samples of this rare disease will require international collaborations [2].

Taken together, our NGS study of OC BM revealed a prominent number of *BRCA*-mutations beside *TP53, ATM* and *CHEK2* mutations. These findings strongly suggest the implication of BRCA and DNA repair malfunction in OC metastasizing to the brain. For any conclusive statement as to whether the DNA damage-recognition and repair pathway plays a key role in this phenotype, the implementation of a similar study with a larger cohort and functional analyses is needed. PARPi represents one of the most promising treatment options for OC patients in general and for OC patients with metastatic BM in particular.

## Electronic supplementary material

Below is the link to the electronic supplementary material.

Supplementary material 1 (DOCX 14 KB)

Supplementary material 2 (XLSX 11 KB)

Supplementary Figure S3. Pie chart of the substitutions revealed in the 8 BM samples sequenced in this study. The majority of the 54 substitutions were transitions (purine–purine and pyrimidine–pyrimidine) (JPG 2430 KB)
