# Supplementary material for: Next-Generation Sequencing-based genomic profiling of brain metastases of primary ovarian cancer identifies high number of BRCA-mutations
Source: J Neurooncol. 2017 May 11;133(3):469–76. doi: 10.1007/s11060-017-2459-z (PMC5537326; doi:10.1007/s11060-017-2459-z)
Supplement: Supplementary file 1 — Supplementary material 1 (DOCX 14 KB) [file 11060_2017_2459_MOESM1_ESM.docx]

**Supplementary Table S1: Overview of patients’ characteristics**

| **Characteristics** | **Entire cohort (n=10)** | |
| --- | --- | --- |
|  | **n** | **%** |
| Median age at diagnosis of ovarian cancer, years  (range) | 51  (36-65) | |
| Stage IV at diagnosis of ovarian cancer |  |  |
| Yes | 5 | 50.0 |
| No | 5 | 50.0 |
| Platinum based chemotherapy before diagnosis of brain metastases |  |  |
| Yes | 9 | 90.0 |
| No | 1 | 10.0 |
| Visceral metastases |  |  |
| Yes | 4 | 40.0 |
| No | 6 | 60.0 |
| Median time from diagnosis of ovarian cancer to diagnosis of brain metastases, months  (range) | 30.5  (0-61) | |
| Number of brain metastases at diagnosis |  |  |
| 1 | 7 | 70.0 |
| 2-3 | 3 | 30.0 |
| >3 | 0 | 0.0 |
| GPA class at diagnosis of brain metastases |  |  |
| Class I | 1 | 10.0 |
| Class II | 1 | 10.0 |
| Class III | 7 | 70.0 |
| Class IV | 1 | 10.0 |
| 1^st^ line treatment for brain metastases |  |  |
| SRS | 1 | 10.0 |
| Surgery | 8 | 80.0 |
| Best supportive cancer | 1 | 10.0 |
| Median survival from diagnosis of brain metastases, months  (range) | 28  (0-166) | |
