# Supplementary figures and images for: Next-Generation Sequencing-based genomic profiling of brain metastases of primary ovarian cancer identifies high number of BRCA-mutations
Source: J Neurooncol. 2017 May 11;133(3):469–76. doi: 10.1007/s11060-017-2459-z (PMC5537326; doi:10.1007/s11060-017-2459-z)

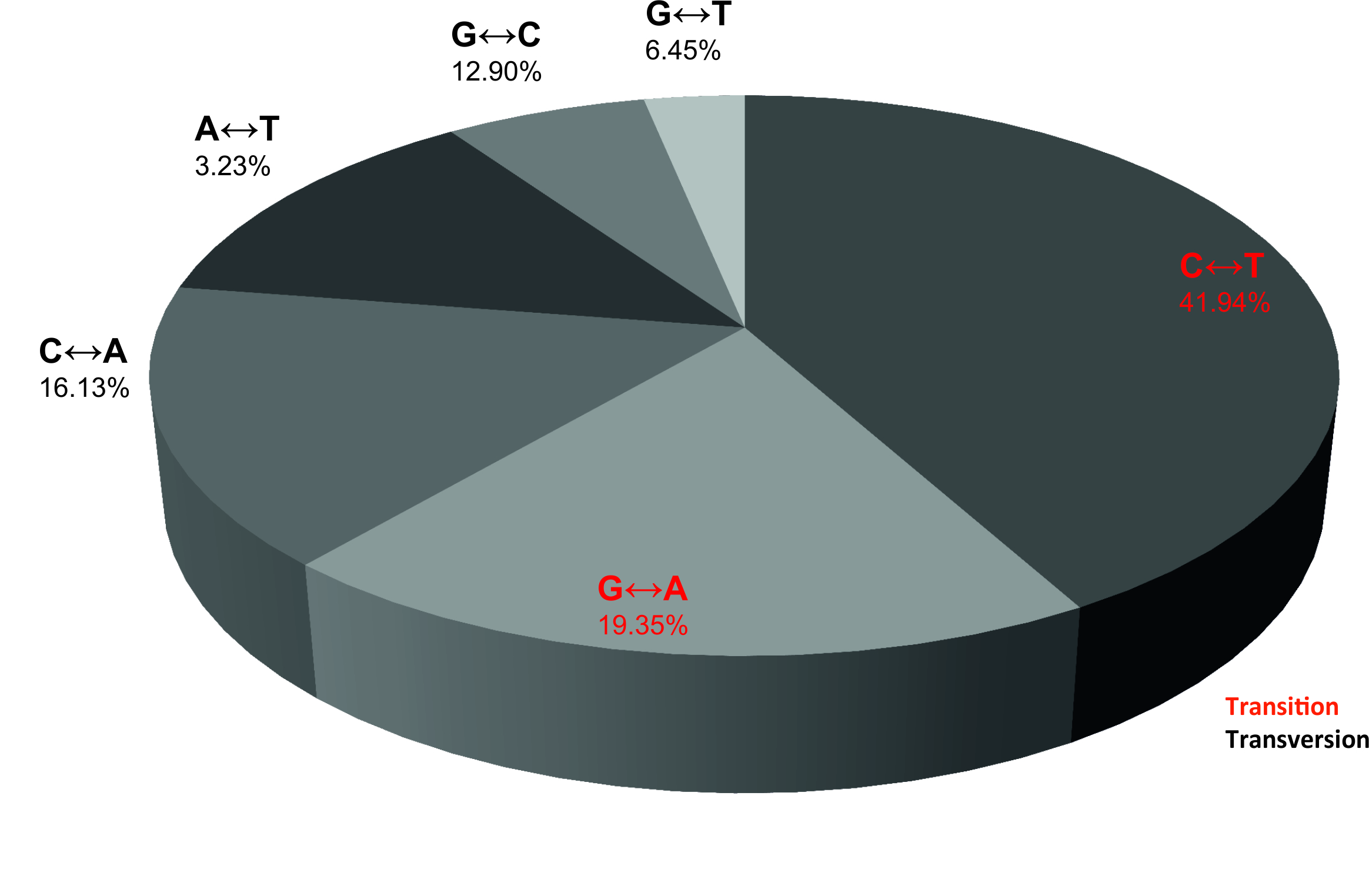

Supplement: Supplementary file 3 — Supplementary Figure S3. Pie chart of the substitutions revealed in the 8 BM samples sequenced in this study. The majority of the 54 substitutions were transitions (purine–purine and pyrimidine–pyrimidine) (JPG 2430 KB) [file 11060_2017_2459_MOESM3_ESM.jpg]
